# Editing the Central Nervous System Through CRISPR/Cas9 Systems

**DOI:** 10.3389/fnmol.2019.00110

**Published:** 2019-05-27

**Authors:** Agustin Cota-Coronado, Néstor Fabián Díaz-Martínez, Eduardo Padilla-Camberos, N. Emmanuel Díaz-Martínez

**Affiliations:** ^1^Biotecnología Médica y Farmacéutica CONACYT, Centro de Investigación y Asistencia en Tecnología y Diseño del Estado de Jalisco (CIATEJ), Guadalajara, Mexico; ^2^Departamento de Fisiología y Desarrollo Celular, Instituto Nacional de Perinatología, Mexico City, Mexico

**Keywords:** CRISPR/Cas9, central nervous system, blood–brain barrier, “Trojan horse” peptides, adenovirus-associated virus, d-Cas9

## Abstract

The translational gap to treatments based on gene therapy has been reduced in recent years because of improvements in gene editing tools, such as the CRISPR/Cas9 system and its variations. This has allowed the development of more precise therapies for neurodegenerative diseases, where access is privileged. As a result, engineering of complexes that can access the central nervous system (CNS) with the least potential inconvenience is fundamental. In this review article, we describe current alternatives to generate systems based on CRISPR/Cas9 that can cross the blood–brain barrier (BBB) and may be used further clinically to improve treatment for neurodegeneration in Parkinson’s and Alzheimer’s disease (AD).

## Introduction

The most common and prevalent worldwide neurodegenerative diseases are Alzheimer’s disease (AD) and Parkinson’s disease (PD). Thus far, there is no cure, nor is the etiology of either condition completely understood. Progression and ultimately death in patients with a very advanced degree of AD/PD may possibly be attributed to not having fully elucidated the molecular mechanisms that underlie these diseases (Kampmann, 2017). However, the CRISPR system and its variations open up a range of unprecedented possibilities for examining the genome and finding potential markers of early expression (Hu et al., 2018) or the development of personalized therapies that have a real impact on neurodegenerative conditions. On the other hand, research groups have developed gene therapy based on editing tools with nucleases by applying ingenious designs *in vivo*, achieving the first cases of clinical patient referrals. These achievements have shown a huge reduction in the marked gap between basic medical research and translational medicine.

**Figure 1:**
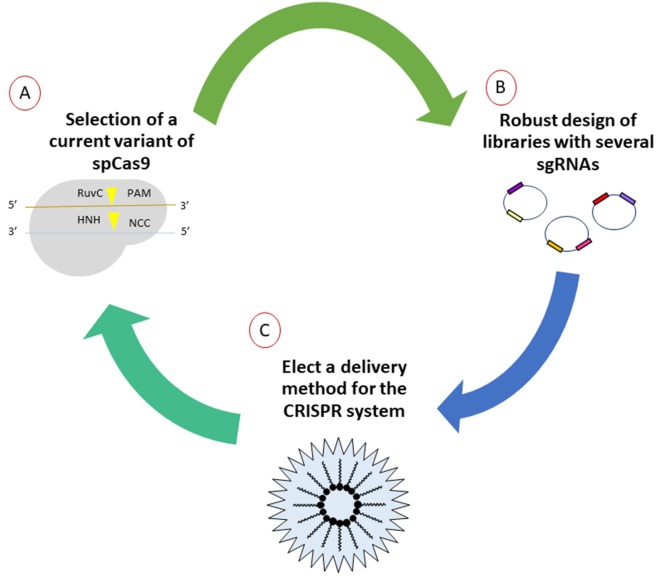
General workflow for generation of CRISPR/Cas9 strategies for purposes of gene therapy. **(A)** First, the mutants or orthologes derived from SpCas9 evidenced by other research groups and those commercially available should be explored. Due to the emerging amount of literature, selection of the most suitable options will impact directly in the cost benefit of editing. **(B)** The elaboration of diverse libraries with thousands of single-guide RNAs (sgRNAs) allows to select those that generate real hits in the flank sequences, lowering the risk of off-target effects, verified by next-generation sequencing later. **(C)** Currently, there are various ways for the efficient delivery *in situ* of gene-editing tools. Several options are currently under investigation, especially methods that involve delivery by means of non-invasive methods.

In order to establish new gene therapies that could be applied in a translational way to neurodegenerative diseases, a robust design approach for gene editing systems must be considered. It should be noted that thorough *in silico* design is the fundamental basis of the performance of therapy *in vivo*, as has been demonstrated through the success of several working groups by monitoring a workflow that is the right choice of the basic elements of the CRISPR/Cas9 system (Figure 1).

## Current Variants of spCas9

Based on previous discoveries, the concept of diversifying the system by looking for other microorganisms with functional orthologous proteins and mutagenesis of the enzyme spCas9 obtained from *Streptococcus pyogenes* was raised in order to increase the percentage of efficiency and delivery when editing. Cebrian-Serrano and Davies (2017) described the structural redesign of spCas9, mutating regions of key residues and the function of the new variants of Cas9 in view of their application in mammalian gene therapy (Cebrian-Serrano and Davies, 2017). Currently, the spCas9 variants successfully used for mammalian gene editing are nCas9, dCas9-FokI, Cas9-VQR, Cas9-EQR, Cas9-VRER, Cas9-D1135E, Cas9-QQR1, SpCas9-HF1, eSpCas9 (1.0), and eSpCas9 (1.1). One of the new systems, referred to as “CRISPR 2.0,” was created by Gaudelli et al. (2017). They managed to perform seventh-generation adenine base (ABEs) editing, allowing the conversion of A-T to G-C in genomic DNA by RNA adenosine deaminase to operate in DNA, through the fusion of a CRISPR-Cas9 mutant to its catalytic site. The results exhibited 50% editing efficiency in human cells with a high purity (99%) and low rates of indels (≤0.01%), producing a more efficient and clean system for the introduction of specific mutations. Remarkably, it allows the direct and programmable introduction of the four mutations, A-T/G-C, without performing the classic rupture in the double-stranded DNA (Gaudelli et al., 2017). Therefore, each system has a specific purpose that is dependent on the nature of the editing to be performed. In the same way, each research group can adapt the fundamental elements of the system into their experiments. Consequently, in a few years, there will be a very diverse toolbox that includes specific Cas9 systems for each human cell lineage (somatic or stem cell), highly efficient in making the most possible changes to the genome without affecting vital functions.

## Robust Design of Single-Guide RNAs

Without doubt, one aspect that directly affects efficiency is the intelligent design of single-guide RNAs (sgRNAs). Previously, the guides were created without consideration for several parameters that have been identified as key factors to increase specificity over time. Several studies have shown that the off-target activity of Cas9 depends on the chemical composition of sgRNA and on the conditions in which the experiment is performed (Fu et al., 2013; Hsu et al., 2013; Ran et al., 2013; Guilinger et al., 2014; Veres et al., 2014). However, some general properties among them are difficult to determine because it is necessary to carry out an extensive series of studies that verify the interaction between DNA and RNA, making it possible to discriminate a true hit from false positives. Therefore, several platforms and algorithms have been established to optimize the generation of very wide libraries of possible sgRNAs for the same gene and are currently available online. Doench et al. (2014) discovered characteristic sequences that improve the activity of sgRNAs and optimize the site PAM for *S. pyogenes*. The study consisted of obtaining a platform that would improve the design of sgRNAs, evaluating a total of 1,841 in mammalian cells, for purposes of genetic screening and editing, culminating in an online tool for the design of highly active sgRNAs useful for any gene^1^.

Based on the results of Doench et al. (2016), human libraries were generated called Avana (110, 257 sgRNAs) and Asiago (120, 453 sgRNAs) selecting six guides per gen, according to the rules to improve the design of sgRNAs (rule set 1; Doench et al., 2014), including specificity within region protein encodings and the localization of the target site within the gene. Using the cutting frequency determination (CFD) score for the most lethal sgRNA shows that a significant increase in the number of off-target sites was found. This observation suggests that guides with previously determined off-target sites exhibit more frequent depletion in negative selection screening, therefore avoiding such promiscuous sgRNAs and resulting in better performance of the designed library (Doench et al., 2016). In previous work, a better online tool was developed, increasing the efficiency in the design of sgRNAs for editing in human and mouse cells; however, the platform only included Cas9 enzymes belonging to *S. pyogenes*, which only recognizes the PAM-NGG site, and Cas from *Staphylococcus aureus*, which recognizes the PAM-NNGRR site. Therefore, it is recommended to use other tools for the design of sgRNAs directed at variants of Cas or Cas9-type enzymes that recognize different sites from PAM of the SpCas9. It should be noted that other research groups have shown that the composition and order of the sequence of the sgRNAs have a direct impact on the success of the editing. For example, Wang et al. (2014) determined that an inadequate design compromises the efficiency and specificity of the desired editing. In another study, it was observed that in the presence of guanine (G) in the first or second position with respect to PAM, the specificity increased. Likewise, Xu et al. (2015) showed that the presence of multiple T adjacent to the PAM site is not advisable because of the loss of specificity. In another work, Moreno-Mateos et al. (2015) observed that sgRNAs rich in G and poor in the presence of adenine (A) promote the increase of stability and efficiency. Another relevant aspect that has been exhaustively assessed is the tolerance of mismatches. It has been reported that there is a greater tolerance for the presence of mismatches in the terminal region of the sgRNAs. Moreover, the presence of five mismatches was defined as the limit of tolerance, because with more than five mismatches, cuts in the sequence at sites where there were no targets and consequently the presence of unwanted mutations were found (Fu et al., 2013). As described above, it is established that by following the above recommendations, large, highly specific, and efficient libraries can be obtained for editing specific target sites and avoiding false positives in gene therapy *in vivo*.

## Improved Methods for the Delivery of CRISPR Components

The advantage of the CRISPR/Cas9 gene editing system in mammalian cells is the high efficiency observed when knock-out models are made by the non-homologous end joining (NHEJ) repair mechanism and the union of nonhomologous ends. However, this efficiency decreases dramatically during the production of knock-in models due to the nature of repairing with the homologous recombination mechanism (HDR; Ran et al., 2013; Rong et al., 2014). Because of the enormous implications of being able to insert fragments at will in the genome in such a specific way, several studies have suggested improvements to increase this repair mechanism. González et al. (2014) co-delivered sgRNA and a single-stranded oligodeoxynucleotide (ssODN) donor in human pluripotent cells (hPSCs) expressing Cas9, generating homozygotic knock-in clones with an efficiency of slightly more than 10%. Lin et al. (2014) synchronized HEK293T cells and hESCs in phase M using the antineoplastic agent nocodazole and introduced nucleofection of the ribonucleoproteins complexes of Cas9 (RNPs) and ssODNs, resulting in a significant increase of the insertion by the HDR mechanism of 38% for HEK293T cells and 1.6% for hESCs, contrasting with negative controls that were not synchronized (26% in HEK293T and 0% in hESCs). Schumann et al. (2015) obtained an efficiency of 20% in the delivery of RNPs-Cas9 and ssODNs in primary T-cells using an electroporation technique, obtaining human knock-in cells with a high efficiency. As previously mentioned, the techniques most used to introduce exogenous material to mammalian cells are lipofection, electroporation, and microinjection. Some of these techniques have been improved with the desire to adapt them to cells that are “tricky” to transform as stem cells. However, to prepare CRISPR technology adequately for use in gene therapy, there are some limitations that have to be overcome (Kelton et al., 2016). Among them is the use of plasmids that contain the Cas9 sequences, usually fused to a nuclear localization (NLS) signal and one or multiple sgRNAs (Sakuma et al., 2014), facilitating the intranuclear expression and securing access to the target site. Despite the remarkable results that have been generated through the use of these all-in-one plasmids, it has been shown that this use can generate constituent expression of Cas9 due to the natural predisposition of the NHEJ repair mechanism and activation of the innate immune system because of the cut double chain (Mandal et al., 2014; Sather et al., 2015). In the same way, several studies have been reported with a therapy-oriented approach where they use some viruses with peculiar characteristics, such as the adeno-associated virus (AAV), which has a packaging capacity of about 4.5 kb and is a non-integrative virus (Senís et al., 2014). However, the number of amino acids that constitute SpCas9 is added to the sgRNA sequence, increasing the total package size. Therefore, it has been determined to divide the system between several plasmids by dividing the enzyme and sgRNAs into their own plasmids (Fine et al., 2015; Wright et al., 2015; Zetsche et al., 2015). This has been found to decrease activity due to the formation of indels after the rupture of the DNA double chain. Due to the disadvantages described, an exhaustive search of ortholog enzymes is being performed for homologies similar to SpCas9 in other microorganisms (Friedland et al., 2015). Ran et al. (2013) reported an isolated Cas9 from *S. aureus* with a size of 3.2 kb and with similar editing efficiency in mammalian cells to native SpCas9, which have 1 kb less information when encoded in the form of DNA. Furthermore, it can be packaged like Cas9 simply within the capsid of the AAV, versifying its application in clinical studies, mainly for the correction of hereditary diseases such as Duchenne muscular dystrophy (DMD), by deletion of the deficient exon to produce the dystrophin protein, a direct cause of this pathology (Nelson et al., 2016).

Another alternative used to reduce the size of Cas9 is the generation of messenger RNA (mRNA) transcript of genes, which has been adapted in embryo editing by microinjection (Wang et al., 2013; Hai et al., 2014; Niu et al., 2014), recently scaling from somatic adult cells (Liang et al., 2015) toward primary cell culture (Osborn et al., 2016) by electroporation. Another advantage of this modality of delivery is the non-integration of the enzyme in the genome. However, there is a lack of conclusive evaluation of factors, such as stability and immunogenic response, to determine whether this method is advantageous over others. Several studies are focusing on the use of lentivirus (LV) because the capsid has the capacity to store around 8 kb, which is more than the capacity of AAV, permitting the expression of Cas9 and sgRNAs at the same time (Kabadi et al., 2014; Sanjana et al., 2014). Their usefulness in the transformation of cells that are not in active division, such as neurons in adults, has also been reported (Blömer et al., 1997; Vezzani, 2007). In addition, it has been shown that gene expression using LVengineering can be sustained for years, without prompting an immune system response from host participants in some clinical trials (Palfi et al., 2014). Due to these favorable properties and the current ineffective treatment, the use of gene therapy using LV has been started in the first clinical trials for the treatment of patients with neurodegenerative disorders of the central nervous system (CNS), opening a promising new stage in the application of molecular therapies that are really effective in improving quality of life, with similar or better results than convectional drugs (Palfi et al., 2014).

## Gene Therapy to Improve Neurodegenerative Process

As previously mentioned, the reason for the search for alternative therapies for treating patients with AD and PD lies in the inefficiency of the current treatment. Yiannopoulou and Papageorgiou (2013) exemplified several drugs recently used for treating AD in clinical trials around the world and directed at three main approaches: (a) anti-platelet agents targeting β-amyloid; (b) selective agents reducing Aβ42; and (c) immunotherapy. However, it has been determined that these disease-modifying drugs exhibit disappointing or doubtful results in phase IIa and III clinical studies (Yiannopoulou and Papageorgiou, 2013). On the other hand, the gold standard treatment for PD patients is the dopamine agonist levodopa (L-DOPA), which can alleviate the consistent symptoms of neurodegeneration for a few years. However, it is well documented that use for prolonged periods has direct consequences on the development of involuntary movements, such as dyskinesias, and behavioral changes (Weintraub et al., 2010). Henceforth, gene therapy is a remarkable option that offers some advantages for the treatment of progressive disorders and its adequate engineering using the CRISPR/Cas9 system displays unique opportunities to eradicate the aberrant genetic components among patients with AD and PD. In addition, the large amount of existing literature is consistent with the need for improving current delivery and release systems that can offer discriminatory properties providing specificity for both the organ and some cell lineages and, in particular, for the integral preservation of the elements that will carry out the specific editing task, regardless of whether they are biological.

## Novel Vehicles and Delivery Methods

**Figure 2:**
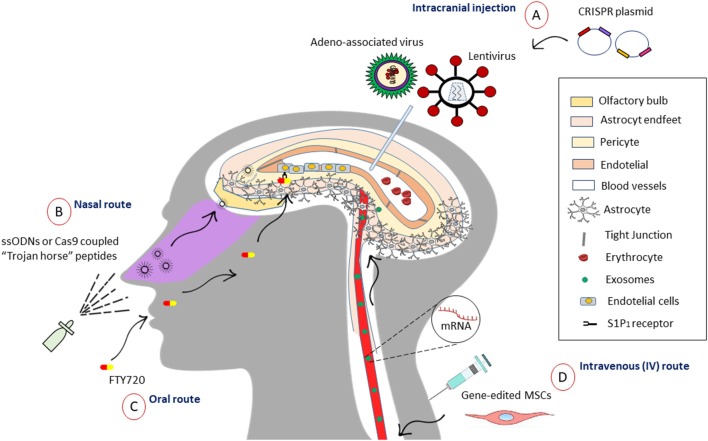
Different strategies to access the central nervous system (CNS). **(A)** Intracranial injection allows the entry of viruses such as the adeno-associated virus (AAV) that can package the sequence of Cas9 and the sgRNA in its capsid to incorporate it in a non-integrative manner. While lentiviruses have eight times the storage capacity of AAVs, so it would be used for the co-expression of Cas9 with distinctive sgRNAs all in one. **(B)** One of noninvasive approaches used to go through BBB is the intranasal administration of “Trojan horse” peptides (5–30 amino acids), which can be coupled to nucleic acids, for example, ssODNs or the enzyme Cas9, facilitating access through the olfactory bulb. **(C)** Oral drugs like FTY720 (Fingolimod) allows inducible opening of the blood–brain barrier (BBB) through sphingosine 1-phosphate receptor-1 (S1P1) receptor in endothelial cells. This may be a promising option to control the temporary access of molecules of 10 kDa or less and larger in the future. **(D)** Mesenchymal stem cells (MSCs), for its homing properties, can travel to the site and perform the repair from the intravenous line to the CNS, acting as vehicles, and it has been shown that they do not arouse meaningful immune responses. Otherwise, the release of small vesicles called exosomes (30–150 nm) allows the transference of mRNA and other regulatory molecules among MCSs, and it has been shown to release them in cells of neural lineage. This can be an advantage for the delivery of Cas9 mRNA and sgRNAs through the BBB.

The main dilemma that arises in designing a therapy directed to the brain is being able to pass through the blood–brain barrier (BBB), which has the function of isolating and protecting neural tissue, controlling the entry of molecules and therefore hindering delivery. About 7,000 drugs have been assessed in the Comprehensive Medical Chemistry database, with only 5% able to cross the BBB to enter the CNS (Pardridge, 2005). Agustín-Pavón and Isalan (2014) illustrated various strategies for the efficient delivery of components to neural tissue (Agustín-Pavón and Isalan, 2014). They divide the methods into two categories: (a) less invasive [intranasal access through nanoparticle (NP)-assisted drug delivery across the BBB is another system with mitigatory properties and the supporting NPs indicate solid colloidal particles with a size range of 1–1,000 nm (Zhou et al., 2018; polymers, lipids, magnetic), emphasizing that successful BBB passage with subsequent cellular labeling could be achieved if NPs were fabricated with non-ionic surfactants or cationic stabilizers but not when anionic compounds were added; in addition, NP’s size (67–464 nm) and charge had no influence on BBB passage (Voigt et al., 2014); among others, they described exosomes, cell-penetrating peptides (CPPs), often vividly termed as “Trojan horse” peptides, or protein transduction domains (PTDs) as a class of diverse peptides, typically with 5–30 amino acids (4–24 nm), that can translate through the cellular plasma membrane (Shi et al., 2014)]; and (b) more invasive (direct injection into the parenchyma of the brain or ventricles during stereotactic surgery, entry of hyperosmotic solutions, microbubbles with ultrasound activation, and laser irradiation (Agustín-Pavón and Isalan, 2014). All these methods can serve as vehicles for the preservation of the integrity of CRISPR systems, improving the possibilities of editing in the target site with the enormous advantage of crossing the BBB. One novel and exciting alternative for accessing the CNS is the reversible and temporal manipulation of entry molecules that access the BBB. A study from Yanagida et al. (2017) showed that pharmacological inhibition (FTY720) and sphingosine 1-phosphate receptor-1 (S1P1) gene facilitates the selective entry of small molecules, opening the BBB, with no major signs of inflammation or CNS damage. This effect was attributed to changes in the cytoskeleton of tight junction proteins. Another pertinent finding was that the pharmacological treatment was reversible and transient, suggesting a viable alternative to intentionally opening the BBB and allowing the entry of molecules of <3–10 kDa (Yanagida et al., 2017). These results suggest another noninvasive alternative to diversifying the available therapeutic options for CNS diseases. On the other hand, subsequent studies could examine the synchronization of FTY720 activity for the introduction of molecules greater than 10 kDa, such as mRNA-Cas9 or ssODNs, for the improvement of their *in situ* action (Figure 2).

Another promising option lies in cell-based therapeutics, adapting mesenchymal stem cells (MSCs) due to their inherent beneficial properties, such as the release of pro-regenerative trophic factors and the generation of bilipid vesicles. These can mediate the transport of small molecules, reported in cell–cell communication and signaling, arousing a great interest as a therapeutic option (Phinney and Pittenger, 2017). Their potential lies in the size of exosomes (30–150 nm) and its form of propagation by means of horizontal transfer of mRNA, micro-RNAs (miRNAs), transfer RNAs (tRNAs), lipids, and proteins, modifying the cellular activities (Sarko and McKinney, 2017). In contrast to release forms, such as liposomes or nanopolymeric particles, exosomes can potentially avoid the endosomic pathway and lysosomal degradation, allowing the release of molecules directly into the cytoplasm (Lou et al., 2017). Likewise, they do not support immunogenic problems observed in other systems of delivery for drugs such as dendrimer (Lee et al., 2005), NPs (Petros and DeSimone, 2010), and liposomes (Torchilin, 2014). Remarkably, it has been observed that these particles can be captured by nearby cells or migrate to distant tissues following circulation in the peripheral blood and through the BBB. Sarko and McKinney (2017) raised the natural reconfiguration of the secretome, allowing the use of exosomes for the controlled release of new drugs and other molecules that act *in situ*. Added to this, it has been demonstrated the release of exosomes in various neural lineages, such as astrocytes, microglia, neurons, oligodendrocytes, and neural stem cells (Sims et al., 2014; Brites and Fernandes, 2015; Budnik et al., 2016; Janas et al., 2016; Verkhratsky et al., 2016), fortifying the idea of reengineering these vesicles for therapeutic purposes in neurodegenerative diseases such as PD and AD. The incorporation of the CRISPR/Cas9 system within these biological vehicles could offer exosomic nanovesicles filled with mRNACas9, ssODNs, or saCas9 with the ability to circulate and access different regions of the brain to edit cells in a niche-specific way, having the advantage of recognizing cellular reception ligands.

Among the other biological vehicles described in the literature, work from Wang et al. (2016) demonstrated the production of self-assembly synthetic lipids, obtained from NPs belonging to bio-reduceable lipids with negatively overloaded proteins, forming Cas9 complexes: anionic sgRNAs for the efficient editing of the genome in mammalian cells and in rodent brains. The correct delivery by stereotactic injection or other noninvasive method could lead to gene correction of mature neurons *in situ*, restoring specific neural circuits through a low-toxicity delivery method (Wang et al., 2016).

Another novel strategy among nonviral approaches for examining the mammalian brain *in vivo* entails coupling the Cas9 enzyme with gold NPs. Lee et al. (2005) demonstrated that gene editing by intracranial injection of CRISPR-Gold NP ribonucleoprotein could be achieved. They performed gene editing in Thy1-YFP and Ai9 mice, in addition to targeting the metabotropic glutamate receptor 5 (*mGluR5*) gene to reduce exaggerated signaling in the striatum of a mouse model of fragile X syndrome (FXS) in a *Fmr1* KO mouse. The team observed gene editing in neurons and non-neuronal cells, including microglia. Remarkably, they inhibited 40%–50% of the expression of the autism-causing gene, *Grm5*, in the striatum after intracranial injection, leading to rescue from autism-associated behavioral phenotypes (Trenkmann, 2018).

In a recent study, Narbute et al. (2019) notably demonstrated for the first time the therapeutic effect of intranasal administration of extracellular vesicles (EVs) derived from human exfoliated deciduous teeth stem cells (SHEDs) in a unilateral 6-OHDA rat model of PD. They succeeded in reversing gait impairments and normalizing TH expression in the substantia nigra and striatum (Narbute et al., 2019). The proteomic assay demonstrates the presence of Cu/Zn superoxide dismutase 1 (SOD1) and antioxidant proteins thioredoxin (TXN) and peroxiredoxin-6 (PRDX6) within the EVs, which the authors suggest may reduce sensitivity of dopaminergic neurons to 6-OHDA-induced oxidative stress based on prior studies (Mazzio et al., 2004; Botella et al., 2008). Nonetheless, little is known about the molecular antioxidant mechanism and further studies are required to unveil the neuroprotective action of EVs, which may be an interesting nonviral approach for treating PD with fewer invasive repercussions.

Despite the novelty and growing interest in noninvasive gene-editing techniques that pass the BBB, more studies of their toxicity and efficiency are needed. In addition, the most recent reports tend to fine-tune control of key pathogenic gene expression; at the moment, this could be achieved through rational engineering-derived viruses such as AAVs, LVs, and retroviruses (RVVs).

## The Growing Promise of Adenoviruses

In recent years, the manufacturing of new AAV serotypes is having an enormous effect on the field of gene therapy, especially in the neurosciences, and as a potential treatment of neurodegenerative diseases (Deverman et al., 2018). Currently, one of the most pursued objectives is the development of more efficient systems for *in vivo* gene editing. Among the most outstanding recent achievements, Suzuki et al. (2016) developed a CRISPR system homology-independent targeted integration (HITI) strategy that enabled knock-in of both dividing and nondividing cells, remarkably in the neurons of postnatal mammals, and demonstrated better efficiency than that of the HDR mechanism. The *in vivo* system consisted of HITI constructs and two AAV vectors and packaged both AAVs with serotype 8 or 9 (AAV-Cas9 and AAV-mTubb3). Afterward, they performed inducible Tubb3–GFP HITI targeting constructs, where Cre-dependent Cas9 expression was under the control of tamoxifen (TAM), by *in utero* electroporation of E15.5 fetal brain. The results revealed efficient GFP knock-in with the HITI donor and minimal knock-in with the HDR donor (Tubb3-HDR).

Furthermore, to validate their therapeutic potential, they demonstrated improvement in visual function in a rat model of retinitis pigmentosa. The *Mertk* gene was corrected using an AAV vector for inserting a copy of *Mertk* exon 2 into intron 1 (AAV-rMertk-HITI; Suzuki et al., 2016). This system promises to be a strong candidate for exploiting the *in vivo* gene-editing approach of the CNS and simultaneously overcoming the drawbacks caused by the use of the HDR repair mechanism.

The other main goal is to perform modifications among serotypes to enable a wider distribution in the large mammalian brain, enabling clinical therapeutic implementation. In another recent work, Naidoo et al. (2018) developed a novel AAV variant (AAV2-HBKO), capable of widespread CNS transduction in neurons localized in the deep cortical layers, deep cerebellar nuclei, several subcortical regions, and motor neuron transduction *via* thalamic injection and intracerebroventricular delivery. The combination resulted in the transduction of oligodendrocytes in superficial cortical layers and neurons in deeper cortical layers. They also demonstrated that the delivery route has an impact on the cellular tropism and pattern of CNS transduction (Naidoo et al., 2018). Interestingly, vectors such as AAV1, AAV5, and AAV9 do not have affinity for heparan sulfate proteoglycans (HSPGs); therefore, they can distribute after a single injection into the brain (Pillay et al., 2016). The AAV2-HBKO serotype has been demonstrated to cope with tropism for HSPGs and showcases in particular different translation patterns in oligodendrocytes and motor neurons, depending on the delivery method of the vector (ICV or thalamic). This poses tremendous implications for providing gene therapy to humans within the next years. Furthermore, it is suitable for neurological disorders that involve many parts of the brain, such as AD, and especially those that involve the hippocampus.

Although it is widely documented that most cases of AD are sporadic, the presence of certain point mutations or deletions in the genes for amyloid precursor protein (APP), presenilin 1 (PSEN1), and presenilin 2 (PSEN2) triggers the production of beta-amyloid peptide. Therefore, the study of the familial component in AD can highlight tremendous opportunities to intervene through gene therapy. György et al. (2018) harvested fibroblasts from carriers of *APPswe* (KM670/671NL APP mutation) indigenous to Sweden (*APPswe* for the mutation and APPSW for the mutant allele) who had increased Aβ levels (Mullan et al., 1992). They mediated a CRISPR/Cas9 knockout of *APP*^SW^ or *APP*^WT^ in the human *APP*^SW/WT^ fibroblasts. Cells treated with Cas9 and sgRNAs showed a robust reduction in unperturbed *APP*^SW^ reads, whereas the relative proportion of *APP*^WT^ reads without indels did not decrease. Thus, CRISPR-induced indels were only detected in *APP*^SW^ alleles and not in *APP*^WT^ alleles when using gRNAs against the mutation (György et al., 2018). Subsequently, they applied the system for *in vivo* disruption of the *APP*^SW^ allele in Tg2576 mice (mice carrying multiple copies of the APPswe mutation). AAV-mediated delivery of SW1 gRNA was applied unilaterally into the hippocampus of adult mutant mice. After 6 weeks, the results confirmed the disruption of the mutation (indels) in primary cortical neurons and in the hippocampus in the APP allele (György et al., 2018). This is the first report of AAV-CRISPR therapy in an animal model of AD. Despite the significant reduction in Aβ40 and Aβ42 in the conditioned media from patient cells treated with gRNA against the *APP*^SW^ allele, in the mouse model, they observed limited gene disruption efficiency as a consequence of the high number of target alleles that was not related to insufficient AAV delivery of CRISPR. Therefore, the assessment of Aβ plaque pathology was not carried out.

The study by György et al. (2018) could lead to new *in vivo* therapeutic approaches based on AAV-CRISPR correction constructs. Regardless, new variants of AAVs with greater penetration and efficiency are still needed.

Other approaches to tackling AD involve targeting the pathological process of chronic neuroinflammation. A study by Raikwar et al. (2019) proposed the glia maturation factor (GMF) as an attractive therapeutic target because of its significant upregulation in various regions of AD brains. They transduced a BV2 microglial cell line with an AAV co-expressing *S. aureus* (Sa)-Cas9 (smaller version of Cas9 than spCas9) and a GMF-specific guide RNA (GMF-sgRNA). They found a few cells expressing SaCas9 while lacking GMF expression, confirming successful GMF gene editing. Nevertheless, they achieved low transduction efficiencies. Therefore, the authors suggest the possibility of exploring transduction efficiencies in embryonic stem cells (ESCs), induced-pluripotent stem cells (iPSCs), primary neurons, astrocytes, and microglia, and also the suitability of tropism-modified AAVs (Raikwar et al., 2019). These results suggest early personalized gene therapy based on Sa-CRISPR-AAVs that includes a novel target and might help to reduce neuroinflammation/neurodegeneration not only in AD but also in PD (Khan et al., 2014).

## Precisely Fine-Tuning Expression Through dCas9 and CRISPRa/CRISPRi Technology

New functions and varieties have been produced since the beginning of the exploitation of native spCas9. Among them, the nickase-dead mutant form of the Cas9 protein (dCas9), fused to a Krueppel-associated box (KRAB) domain, could achieve precise and programmed transcription activation and repression, epigenetic remodulations of local histones, DNA modifications, labeling of the genomic locus, and single-base genome mutagenesis (Dominguez et al., 2016; Nishida et al., 2016). This, therefore, represents an attractive method of performing inducible repression of target loci in neuronal cells that overexpress pathological genes when utilizing dCas9-CRISPR interference (CRISPRi) technology and may decrease toxic levels of proteins *via* fine-tunable knock-down. Recently, Zheng et al. (2018) developed two conditional CRISPRi tools to silence *Syt1* expression in either glutamatergic or GABAergic neurons with dCas9-KRAB expression under the control of pCaMKIIα and pVGAT promoters, respectively. They stereotactically infused LV encoding the *Syt1-targeted* conditional CRISPRi system into the mouse dentate gyrus (DG). They observed highly specific enrichment of the genes encoding vesicular glutamate transporter 1 (Vglut1) in pCaMKIIα::dCas9-KRAB+ neurons and glutamic acid decarboxylase 1 (Gad1) in pVGAT::dCas9-KRAB+ neurons. Remarkably, they found subtype-specific expression of the CRISPRi in the DG. Furthermore, *Syt1* expression was selectively abrogated by conditional CRISPRi within the *Syt1-targeting* glutamatergic or GABAergic neurons but not in both, emphasizing the versatility of efficient disruption in a specific subtype of neurons in living mammals (Zheng et al., 2018).

As mentioned previously, a potential strategy could be the implementation of dCas9-CRISPRi intervention to repress the levels of abnormal multiple copies of well-known pathological genes, such as *SNCA* in PD, in a reversible way. Heman-Ackah et al. (2016) demonstrated the utilization of CRISPRi as a robust tool for significantly repressing the expression *via* the transcription start site (TSS) of multiple genes involved in proteinopathy-induced neurodegeneration. They transfected HEK293T simultaneously with dCas9 sgRNAs targeting the alpha-synuclein, microtubule-associated protein tau, APP, and huntingtin (*SNCA*, *MAPT*, *APP*, and *HTT*, respectively). Notably, they performed precise transcriptional modulation through CRISPRa of neurodegenerative disease-related genes in human iPSC-derived neurons. TSS2-2 sgRNA and dCas9-VPR transcriptional activator mediated the activation of alpha-synuclein in normal alpha-synuclein levels (NAS) iPSC-derived neurons from healthy control patient and iPSCs derived from a patient with PD caused by alpha-synuclein triplication (AST). They achieved an eightfold activation of endogenous SNCA expression in the NAS iPSC-derived neurons and through dCas9-KRAB repression, a 40% reduction in alpha-synuclein mRNA levels in the AST iPSC-derived neurons (Heman-Ackah et al., 2016). In addition, the group found that targeting the genes close to the TSS region reduced the off-target effects considerably (Heman-Ackah et al., 2016), reducing the negative side effects of using SpCas9. Overall, these findings suggest the possibility of exploiting the tunable CRISPRa/CRISPRi platform for multiplex transcriptional repression of molecular pathological signatures *in vivo* in the mammalian brain and provide enormous possibilities for addressing neurodegeneration in the familial and sporadic disease states.

## Suitability for Gene-Editing Technology in Parkinson’s Disease and Alzheimer’s Disease?

It is well known that the sporadic component is dominant in the causality of the most common neurodegenerative diseases; however, in recent years, the relevance of several genes and their participation in triggering of the pathological process have been demonstrated. Zafar et al. (2018) followed the descendant population carriers of the “Iowa-Kindred” mutation in PD and showed that triplication in the *SNCA* gene on one allele directly increased alpha-synuclein levels two-fold and lead to a rapid progression of synucleopathy, causing severe clinical and neuropathological features (Zafar et al., 2018). Therefore, the pursuit of restoring normal transcript levels of *SNCA* could have a deep clinical impact. Regarding this approach, as stated above, Heman-Ackah et al. (2016) implemented an iCRISPR-based platform to tackle this in hiPSCs and also the group of Kantor et al. (2018) recently developed an epigenetic-based approach. Using an “all-in-one” lentiviral vector encoding sgRNA-dCas9-DMNT3A directed to intron 1 hypermethylation, strikingly they restored normal levels of *SNCA* mRNA and efficiently transduced hiPSC-derived dopaminergic neurons, resulting in an effective and targeted modification of the methylation state of CpGs within *SNCA* intron 1 (Kantor et al., 2018). Taking into consideration that α-synuclein immunohistochemistry is currently the gold standard in the neuropathological evaluation of PD (Stefanis, 2012), the development of personalized treatments to reduce the levels of both SNCA and α-synuclein is imperative and now achievable. These examples are promising and demonstrate the suitability to perform CRISPR gene-editing approaches in PD, but much more has to be done toward clinical applications.

On the other hand, unfortunately in AD, multiple phase III clinical trials have failed to recover memory and cognitive function in AD patients using trial drugs, such as anti-Aβ antibody; therefore, gene therapy is an interesting option that is being supported by a few recent works. It is well known that a very small percentage of cases (<1%) are caused by known mutations in the APP protein or gene products involved in processing APP to form beta-amyloid. Despite the small percentage of cases caused by these mutations, they all trigger the production of beta-amyloid peptide (Bettens et al., 2013; Rohn et al., 2018). Notably, it has been demonstrated that they can further influence the appearance of early-onset AD before the age of 60. Among the targets described are PSEN1 and PSEN2 genes. To probe the feasibility of the CRISPR approach in AD, Ortiz-Virumbrales et al. (2017) generated human basal forebrain cholinergic neurons (BFCNs) *in vitro* from hiPSCs harboring the PSEN2^N141I^ mutation, based on evidence indicating this population as one of the first cell types to be affected in all forms of AD, and that their dysfunction is clinically correlated with impaired short-term memory formation and retrieval. Interestingly, the CRISPR/Cas9 correction of the PSEN2 point mutation abolished the electrophysiological deficit, restoring both the maximal number of spikes and spike height to levels recorded in healthy controls (Ortiz-Virumbrales et al., 2017). The authors suggest that accumulation of Aβ could synergize with the altered electrophysiological mechanisms in a pathway leading to AD, but the exact mechanism and pathways remain unknown. In another exciting study, Park et al. (2019) targeted beta-secretase 1 “*Bace1*” gene (which is required for the production of Aβ peptides) through CRISPR/Cas9-loaded nanocomplexes in post-mitotic neurons *in vivo* and demonstrated their therapeutic application in five familial AD (5XFAD) and APP knock-in AD mouse models. In the present study, despite not being a viral vector-based delivery, it was nonetheless shown to have a high editing efficiency, thus highlighting the emerging technology of nonviral NPs as an interesting therapeutic option for AD. Despite that CRISPR-based gene-editing and gene-therapy approaches have been seen as powerful tools to restore monogenic diseases, new approaches have provided a compelling basis to suggest the possibility of application to the sporadic component as well. Sun et al. (2018) published in a pre-print that editing of endogenous APP at the extreme C-terminus and reciprocal manipulation of the amyloid pathway through an AAV9-APP gRNA-GFP leads to attenuation of β-cleavage and Aβ while up-regulating neuroprotective α-cleavage. These findings suggest a robust gene-editing approach in human cell lines and *in vivo* in mice^2^.

Overall, although there are only a handful of recent works, they represent significant advances toward the development of more specific therapies for halting neurodegeneration in AD and PD. The most outstanding is the capacity to apply CRISPR-AAV/NP systems in the sporadic group of these diseases. With refinement of some of the current hurdles for clinical application, there is the potential for a tremendous advance for the treatment of neurodegenerative diseases, starting with the most prevalent worldwide.

## Drawbacks to Overcome

It has been demonstrated in the most recent works that a huge benefit of the implementation of treatments based on gene therapy is to cope with the drawbacks of conventional therapeutics and avoid or decrease the side effects that they cause. The age of gene therapy experienced exponential growth as a result of the adaptation of the CRISPR systems for genome-editing in mammals. However, there are still relevant drawbacks that need to be overcome before their clinical implementation in the treatment of neurodegenerative diseases. First, we have to emphasize the issue of the immune response. After the initial “CRISPR fever,” there have been significant questions over the last 2 years regarding the activation of the immune response as a result of the presence of exogenous proteins such as Cas9 and their orthologs. The study from Charlesworth et al. (2019) showed the presence of humoral response and specific antigen T-cells against SaCas9 (Cas-derivative of *S. aureus*) in healthy human volunteers, presenting interesting data for the discussion of the implementation of gene therapies clinically. Nonetheless, both studies call on the international community of researchers to exercise a more rigorous assessment of the possible immunologic responses to the microbial origin of the system (Charlesworth et al., 2019).

As a way of counteracting some of the pitfalls, CRISPR/Cas9-based CNS-targeted therapies are currently being refined to increase specificity for target organs. An example is a work published by Murlidharan et al. (2016). They developed a chimeric AAV2, AAV2G9, improving the activity of AAV2 and AAV9, for the transduction of neurons within the brain, reducing glial infection (Murlidharan et al., 2016). It was also shown that a single administration of cerebrospinal fluid did not lead to the presence of the synthetic virus in off-target organs. In addition, a single intracranial injection with a vector that encodes for the gene *MIR137* (a risk gene for schizophrenia) resulted in the specific deletion of the same gene in the brain of knock-in mice, without detection of the vector in the liver (Murlidharan et al., 2016). An interesting finding of this study was the preferential neuronal tropism of AAV2g9, attributed to the presence of a greater concentration of HSPGs on the surface of the neurons greater than that of glial cells (Hsueh and Sheng, 1999). In this way, the restriction of editing to the neural lineage within the CNS is being carried out in almost exclusively, overcoming the disadvantages regarding selectivity of target organs presented by other serotypes of AAV, also demonstrated in the study mentioned for the AAV9 serotype.

Another important consideration for AAV serotypes in CNS gene therapies was addressed in a study by Long et al., where it was reported that intra-muscular, intra-peritoneal, and retro-orbital delivery form of AAV9 could not pass through the BBB in order to restore the dystrophin protein (expressed in cardiac muscle and skeletal) in the CA1/CA2 regions of the hippocampus (Long et al., 2016). Therefore, it is not possible to cross the BBB for any AAV serotype if stereotactic surgery is not performed in any specific region of the brain. In another study, Swiech et al. (2015) reported the interrogation of the genome *in vivo* by AAV containing SpCas9-sgRNAs applied *via* stereotactic injection, having *MECP2*, *DNMT1*, *Dnmt3a*, and *Dnmt3b* as target genes in the adult mice brain, efficiently achieving multiple-gene editing of post-mitotic neurons in the visual cortex.

These studies agree that with the repeated administration of doses of AAV, concomitant disturbance caused by stereotactic surgery for effective infection and the proper distribution of the virus in the different regions of the brain are necessary. Despite the low risk present during surgery, an insult is consistently generated with the perfusion of the BBB, which could increase the release of inflammatory factors, such as IFN-γ and TLR4, coupled with the inherent reaction of the glia reactive inherent, observed in disorders such as PD and AD (Booth et al., 2017; Wood, 2017). However, this same condition of the BBB is compromised by inflammation and has been shown to allow the transfer of both small and large molecules, participating as a passive mechanism for the transport of drugs to the CNS (Shlosberg et al., 2010). In addition, due to the progressive nature of PD and AD, changes in the function of age-dependent microglia have been shown. Spittau described these changes at the molecular level, including the TLR-dependent triggering of alpha-synuclein (αSyn), activation of microglia through neuromelanin, and αSyn deficient phagocytosis in PD, whereas in AD, the decrease in phagocytosis of the β-amyloid plaques (Aβ) and depletion of the microglia do not decrease the formation of plaques and neurodegeneration continues (Spittau, 2017). As described above, gene manipulation of the microglia could have interesting results in mediating inflammation or reducing hyperreactivity in the same way that it could be used in different CRISPR systems, such as dCas9 for the regulation of the release of trophic factors. On the other hand, it has been shown that the R47H mutation of the *TREM2* gene can cause a three- to four-fold increase in the risk of developing AD in a 5XFAD murine model (Wang et al., 2015). Wang et al. (2015) showed that TREM2 deficiency and haploinsufficiency increase the accumulation of β-amyloid (Aβ) due to a dysfunctional microglial response, which fails in clustering around the Aβ plaques and becomes apoptotic. However, more studies are needed to elucidate concisely the role of TREM2 in the development of AD and to evaluate the therapeutic potential of editing its gene variants. Therefore, the dissection of signaling pathways affected by variants will be essential for the design of gene-editing strategies to restore functionality *in situ*.

One of the great advantages that have been explored by some research groups is the performance of multiplex gene editing and the usefulness that CRISPR systems exhibit relative to other nucleases. Cong et al. (2013) first demonstrated the efficient editing of mammalian and human cells simultaneously; therefore, the versatility to program the enzyme and its guides was proposed, with the purpose of recognizing more regions of the genome, taking away the restriction of only being able to access every 8 bp for the SpCas9, a CRISPR system type II. Tothova et al. (2017) achieved multiple edits of CD34+ human hematopoietic stem cells from the umbilical cord and adult primary progenitor cells, which give rise to pre-malignant myeloid and malignant diseases, recognizing the difficulty of modeling these conditions in human cells. Other strategies highlight multiple edits at the epigenetic remodeling level. Savell and Day (2017) exemplify modular approaches for specific targeting and modification of the local chromatin environment at a single gene within the CNS across CRISPR/Cas9 systems. Moreover, they described the purpose of intervening in the regulation of crucial mechanisms that are affected in neuropsychiatric and neurologic disorders (Savell and Day, 2017). Morita et al. (2016) adapted dCas9-SunTag to demethylate a specific locus of DNA, demonstrating the demethylation of CpGs in regulatory regions of ESCs, cancer cells, and primary neural precursor cells and in mouse fetuses *in vivo*. This was the first report of epigenomic manipulation performed *in vivo* based on CRISPR technology and should promote gene modification in living organisms, including modifications in the CNS.

## Conclusions and Perspectives

The recent findings indicate that temporal and region-specific editing of gene expression *via* nonviral and viral approaches throughout the large mammalian brain *in vivo* is achievable. Nevertheless, error-prone mechanisms remain dominant after CRISPR/Cas9 execution; in the adult brain, it is virtually impossible for post-mitotic neurons to utilize the HDR mechanism efficiently for ssODN replacement integration. Therefore, the refinement of new alternatives, such as HITI (Suzuki et al., 2016), will be necessary to ensure therapeutic potential in nondividing cells. On the other hand, a new wave of nonviral delivery systems is approaching. Coupling with stable RNP complexes will allow remarkably novel ways to treat neurological disorders without the need for such invasive procedures. However, more in-depth studies are needed to elucidate its true toxicity and efficiency over prolonged periods (Table 1).

**Table 1:** Novel nonviral and viral systems for *in vivo* gene therapy in the mammalian brain.

	Model	Efficiency	Toxicity	Route	References
**Nonviral**					
*Bio-reduceable lipids nanoparticles*	Rosa26^tdTomato^ mouse	Highly protein delivery but minimal diffusion	Low	DM, DG, MD, cortex, BNST, LSV, paraventricular nucleus of hypothalamus (PVN), and lateral hypothalamus (LH)	Wang et al. (2016)
*Complexes Ribonucleoprotein CRISPR:Gold nanoparticles*	Thy1-YFP, Ai9 and *Fmr1* KO adult mice	High	Potentially accumulate but tolerated in the brain	Intracranial injection in the striatum	Lee et al. (2005)
*dCas9-Suntag-TET1CD All-in-one vector*	Mouse fetal brain at E14	High	No significant off-targets	*In utero* electroporation into VZ	Morita et al. (2016)
*Extracellular vesicles (EVs) derived from SHEDs*	Unilateral rat PD model of 6-OHDA	High	None	Intranasal administration	Narbute et al. (2019)
**Viral**					
*AVV serotypes 8 and 9 (HITI)*	Fetal brain and adult mouse model of retinitis pigmentosa	Low, partial recovery of vision	High on-target specificity of HITI (90%–95%)	*In utero* electroporation and direct injection in the visual cortex	Suzuki et al. (2016)
*AAV2-HBKO serotype*	Adult male *Rhesus macaques*	High number of transduced motor neurons	Perivascular cuffing and cellular infiltration in the thalamus but none presented any gross adverse clinical signs	Bilaterally thalamic injection	Naidoo et al. (2018)
*Cas9/gRNA into exo-AAV1*	Tg2576 mice	Insufficient AVV-delivery of CRISPR	Non-assessed	Direct injection in the hippocampus	György et al. (2018)
*LVVs d-Cas9-KRAB*	Mice with *Syt1* deficiency	Very high	Minimal off-target effects	Stereotactically in the DG	Zheng et al. (2018)

*Currently, rationally engineered viruses are the most suitable approach for selective neural gene-therapy replacement in mammals; however, the efficiency and off-target effects of CRISPR-Cas variants still need to improve before use in clinical scenarios. Therefore, manufacturing new AAV serotypes that can easily cross the BBB and distribute broadly with a single injection will be an intense area of research in molecular neuroscience in the following years. Hopefully, identification of successful doses pre-clinically and maturation of multiplex d-Cas9 AAV-CRISPRa/CRISPRi platforms will support the introduction of carefully regulated gene editing/gene therapy to the treatment of PD and AD patients*.

## Author Contributions

AC-C made a substantial, direct, and intellectual contribution to the work. ND made an intellectual contribution and conducted manuscript review. EP-C made direct intellectual contribution and final approval to the manuscript. ED-M made a substantial intellectual contribution and performed the final revision of the manuscript.

## Conflict of Interest Statement

The authors declare that the research was conducted in the absence of any commercial or financial relationships that could be construed as a potential conflict of interest.

## Footnotes

^1^https://portals.broadinstitute.org/gpp/public/analysis-tools/sgrna-design

^2^https://www.biorxiv.org/content/biorxiv/early/2018/04/28/310193
